# Radio Frequency Drying Behavior in Porous Media: A Case Study of Potato Cube with Computer Modeling

**DOI:** 10.3390/foods11203279

**Published:** 2022-10-20

**Authors:** Xiangqing Chen, Yu Liu, Ruyi Zhang, Huacheng Zhu, Feng Li, Deyong Yang, Yang Jiao

**Affiliations:** 1 College of Food Science and Technology, Shanghai Ocean University, Shanghai 201306, China; 2 Engineering Research Center of Food Thermal-Processing Technology, Shanghai Ocean University, Shanghai 201306, China; 3 College of Electronics and Information Engineering, Sichuan University, Chengdu 610065, China; 4 College of Engineering, China Agricultural University, Beijing 100083, China

**Keywords:** radio frequency drying, finite element method, heat transfer, mass transfer, water vapor concentration

## Abstract

To study the mechanism of heat and mass transfer in porous food material and explore its coupling effect in radio frequency (RF) drying processes, experiments were conducted with potato cubes subjected to RF drying. COMSOL Multiphysics^®^ package was used to establish a numerical model to simulate the heat and mass transfer process in the potato cube and solved with finite element method. Temperature history at the sample center and the heating pattern after drying was validated with experiment in a 27.12 MHz RF heating system. Results showed the simulation results were in agreement with experiments. Furthermore, the temperature distribution and water vapor concentration distribution were correspondent with water distribution in the sample after RF drying. The water concentration within the food volume was non-uniform with a higher water concentration than the corner, the maximum difference of which was 0.03 g·cm^−3^. The distribution of water vapor concentration in the sample was similar to that of water content distribution since a pressure gradient from center to corner allowed the mass transfer from the sample to the surrounding in the drying process. In general, the moisture distribution in the sample affected the temperature and water vapor concentration distribution since the dielectric properties of the sample were mainly dependent on its moisture content during a drying process. This study reveals the mechanism of RF drying of porous media and provides an effective approach for analyzing and optimizing the RF drying process.

## 1. Introduction

Drying is one of the oldest food-preservation processes, which reduces water content and water activity to prolong food shelf-life [1]. Hot air drying is the most popular drying method because of its wide applicability and low cost. However, one of its disadvantages is that heat transfer is from the outer surface to the center of sample, which is in the opposite direction of mass transfer. This usually results in surface hardening, lowering the mass transfer rate and product quality [2]. Novel drying technologies are still needed due to the consumers’ demand for a high-quality food product, which retains most of its original color, flavor, and nutrients, with a higher rehydration rate, better appearance, and moisture uniformity. Moreover, food manufacturers are continuously searching for high-efficiency and energy-saving drying technologies and equipment [3].

Recently, radio frequency (RF)-assisted vacuum drying, heat pump drying, and hot air drying were explored and showed a significant effect [4,5,6]. Previous studies have demonstrated that the application of RF heating technology in drying could shorten drying time and maintain a good quality of products. Wang et al. [7] studied the effects of carrot slices in hot-air-assisted RF drying. Results showed hot-air-assisted RF drying reduced 30% of the drying time compared to solely hot air drying and maintained the good quality of carrot slices. Zhang et al. [8] adopted a two-stage drying method to treat mango slices, which utilized hot air to dry mango slices to 40% (w.b.) in the first stage and then used hot-air-assisted RF heating (HA-RF) to continue to dry the product to 18% (w.b.). Results demonstrated that the developed two-stage drying process took less time (5 h) compared to vacuum drying (7 h) and hot air drying (8 h) and maintained good product quality.

Various factors influence the electric field distribution and the temperature distribution in the product during RF drying, including the shape and size of the sample, electrode gaps, and the properties of materials, etc. [9,10]. The uneven distribution of temperature significantly affects the moisture distribution and ultimately affects the product quality [11]. In RF drying experiments, it is difficult to visualize the effects of different factors on the interior of the product, such as electric field, temperature, water concentration, and water vapor concentration [12]. In addition, experimental approaches have some disadvantages, such as long cycles, high economic cost, low efficiency, and operating difficulties. Compared to the experimental method, computer simulation is an economical, flexible, and intuitive way to reveal the invisible parameters of products in a complex drying process. Computer simulation has already been proven to be effective in predicting and optimizing parameters in RF heating processes [13]. Therefore, the establishment of a mathematical model of RF drying performance of porous material based on multiphase simultaneous transportation is of great significance to simulate the RF drying performance on food products.

The RF drying process couples heat, mass, and electromagnetic energy conversion to heat. Additionally, many foods are plant-based, with intracellular and intercellular water, which adds complexity to the system description. Analyzing the combined effects would assist in fully understanding the RF drying mechanism and facilitating the drying process design. Jia et al. [14] developed a one-dimensional mathematical model to describe the transport phenomena of wood during continuous RF combined with vacuum drying. Their study suggested that the dry zone traveled from the end to the center of the wood and the temperature within the wood decreased with water loss. Hou et al. [15] developed a 3D multiphase porous model to simulate heat and mass transfer of kiwifruit slices under RF–vacuum drying with COMSOL Multiphysics^®^. The results of the simulation agreed well with those of the experiment. The sample temperature at the center was below that at the corners and edges, and the lowest temperature was at the center for a single kiwifruit slice. However, the distribution of moisture content was opposite to that of temperature. The drying rate increased rapidly and then decreased slowly until the end. However, there has not been a 3D numerical model established to comprehensively visualize the distribution of electromagnetic field, moisture, vapor concentration, and temperature within food samples during RF drying at atmospheric pressure. To promote the application of RF drying technology in the food-processing industry, there is a significant need to establish a 3D multi-phase porous model to assist food-processing protocol development. In this study, a mathematical model was established with COMSOL Multiphysics^®^ software package to analyze the temperature, moisture, and vapor concentration distribution within a potato cube as a model food throughout the RF drying process.

The specific objectives of this study were: (1) to develop and solve a fundamental-based mathematical model for an RF drying process with an example sample of potato cube; (2) to validate the model with an experiment by comparing the temperature–time history, surface temperature distribution, and water content with modeling results; and (3) to predict the electromagnetic field intensity, moisture distribution, vapor concentration, and temperature distribution within samples during RF drying.

## 2. Materials and Methods

### 2.1. Model Development

Basic assumptions:

Within all the domains in the model:

(1). All three phases (gas, liquid, solid) in the food sample were continuous media;
(2). All gases were ideal gases;
(3). All fluid, including gas and liquid, were with the same pressure;
(4). Evaporation and condensation occurred within the whole food matrix rather than only on the surface;
(5). Volume shrinkage was not considered; i.e., the food porosity was set as a constant;
(6). Gravity was neglected.

**Figure 1:**
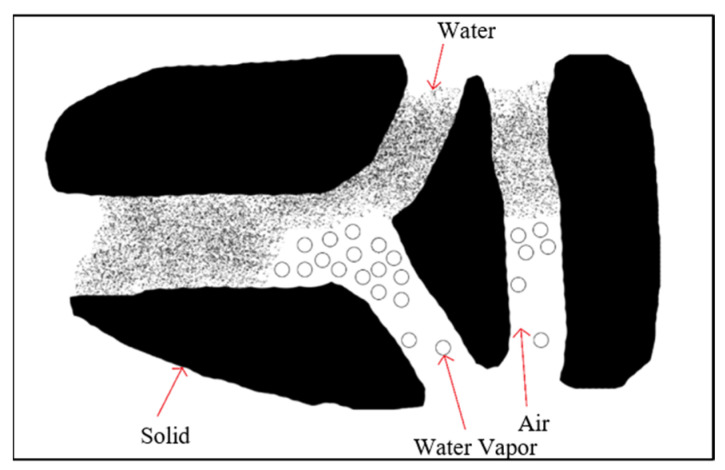
Structural scheme of porous media.

Definition of components in a porous medium:

In a potato cube sample, the internal structure was described as a porous medium as shown in Figure 1, with solid (*s*), water (*w*), and gas (*g*) phases. The gas phase include both water vapor (*v*) and air (*a*), which were both fluid (*f*). The volumetric fraction of solid and fluid in the sample was expressed as [16]:

(1) $$\Delta V=\Delta V_{s}+\Delta V_{f}$$

where *V* is the volume of the potato sample (m^3^), *V_s_* is the volume of solid (m^3^), and *V_f_* is the volume of fluid (m^3^). The porosity of the porous medium can be defined as the volume fraction occupied by fluid, which was expressed as [17]:

(2) $$\emptyset =\frac{\Delta V_{f}}{\Delta V}=\frac{\Delta V_{w}+\Delta V_{g}}{\Delta V}$$

where ∅ is the porosity (–). The volumetric concentration of different phases is related to its saturation, which was expressed as [16]:

(3) $$S_{i}=\frac{\Delta V_{i}}{\Delta V_{p}}=\frac{\Delta V_{i}}{\emptyset \Delta V}\;i=w,\;g$$

where *S_i_* is the saturation ratio (–), and *V_p_* is the volume of the pore (m^3^).

#### 2.1.1. Governing Equations

Maxwell equations are the fundamental base of electromagnetic wave propagation and electromagnetic energy conversion. Since the magnetic properties in foods are usually negligible, and the energy conversion from electromagnetic energy to heat is much faster than the speed of heat transfer, Laplace’s equation can be used to describe the energy conversion in RF heating [18]:

(4) $$-\nabla \cdot (\left(\sigma +j2\pi f\varepsilon_{0}\varepsilon^{\prime }\right)\nabla V)=0$$

where *σ* is the electrical conductivity of the food material (S·m^−1^); *j* is the imaginary part of the square root of −1; $j=\sqrt{-1}$; *ε*_0_ is the permittivity of electromagnetic waves in free space, which is 8.854 × 10^−12^ F·m^−1^; *ε′* is the relative dielectric constant of the food material (–); and *V* is the electric potential across the electrode gap (V).

The power conversion from electromagnetic energy to heat is described by the following equation [19]:

(5) $$Q=2\pi f\varepsilon_{0}\varepsilon^{\prime \prime }{\left|\vec{E}\right|}^{2}$$

where *Q* is the electromagnetic power conversion in the sample to be heated (food) per unit volume (W·m^−3^), *f* is the working frequency of the RF equipment (Hz), *ε″* is the relative loss factor of the food material (–), and $\vec{E}$ is the electric field intensity in the food material (V·m^−1^).

#### 2.1.2. Mass Transfer

The concentration of liquid water, water vapor, and gas can be estimated with the microscopic mass balance of different liquid phases as [16]:

(6) $$\frac{\partial c_{i}}{\partial t}+\bar{\nabla }\;{\bar{n}}_{i}=-\dot{I}$$

i=w, g, v

where *c* is the concentration of component (mol·m^−3^), *n* is the mass flux per unit volume (mol·m^−3^·s^−1^), and *İ* is the rate of evaporation or condensation due to phase change (mol·m^−3^·s^−1^). The liquid water in the pores of food has capillary force, which is in an opposite direction to the internal pressure and hinders the water migration. Thus, the net pressure *P_w_* on the liquid water can be expressed as [16]:

(7) $$P_{w\;}{=P\;-\;P}_{c}$$

where *P_w_* is the capillary pressure (Pa), *P* is vapor pressure (Pa), and *P_c_* is net pressure (Pa).

Water flux (${\bar{n}}_{w}$) is expressed as follows based on Darcy’s law [16]:

(8) $${\bar{n}}_{w}\;{=-\rho }_{w}\frac{k_{in,w}k_{r,w}}{\mu_{w}}\nabla P_{w}{=\rho }_{w}\frac{k_{in,w}k_{r,w}}{\mu_{w}}\nabla {P+D}_{w,\;cap}\nabla c_{w}$$

where *D_w,cap_* is the capillary diffusion rate (m^3^·s^−1^), $D_{w,\;cap}=\rho_{w}\frac{k_{in,w}k_{r,w}}{\mu_{w}}\cdot \frac{\partial P_{c}}{\partial c_{w}}$, *k_in_* is the intrinsic permeability (m^2^), and *k_r_* is the relative permeability. The gas flux (${\bar{n}}_{g}$), including air and water vapor, can be obtained by Darcy’s law [16]:

(9) $${\bar{n}}_{g\;}{=-\rho }_{g}\frac{k_{in,g}k_{r,g}}{\mu_{g}}\nabla P$$

The mass flux of water vapor (${\bar{n}}_{v}$) is dominated by both the pressure-driven mass flow and the diffusivity of the air at the boundary [20]:

(10) $${\bar{n}}_{v\;}{=-\rho }_{v}{\bar{v}}_{g}-\left(\frac{C_{g}^{2}}{\rho_{g}}\right)M_{v}M_{a}D_{bin}\nabla x_{v}$$

where *M* is the molecular mass, and *C_g_* is the mass fraction.

The mass fraction of water vapor can be obtained from water vapor mass conservation and converted to water vapor concentration (*c_v_*). Thus, the gas concentration (*c_g_*) can be determined with the following equations [16]:

$$c_{v}{=w}_{v}c_{g}$$

$$w_{a}{=1-w}_{v}$$

(11) $$c_{a}{=w}_{a}c_{g}$$

The phase changes in a drying process, including the water evaporation and condensation, due to the high pressure generation within the food matrix during drying can be represented as [21]:

(12) $$\dot{I}{=K}_{evap}\frac{M_{v}}{RT}{(P}_{v,eq}{-\;P}_{v}{)S}_{g}\emptyset$$

where *P_v,eq_* is the equilibrium pressure of water vapor in food (Pa), *P_v_* is the calculated instant water vapor pressure from Darcy’s law (Pa), *M_v_* is molecular mass of water vapor, *R* is universal gas constant (kJ·kmol^−1^·K), and *K_evap_* is an evaporation rate constant (1000 s^−1^) [22].

#### 2.1.3. Momentum Balance

During drying, the permeability of water and gas in porous media is relatively low, and thus, the momentum conservation can be expressed by Darcy’s law. The pressure gradient is the main driven force of water and gas transport in a drying process. The diffusion rate of water and gas calculated following Darcy’s law and pressure gradient is expressed as [16]:

(13) $${\bar{n}}_{i}=-\frac{k_{in,i}k_{r,i}}{\mu_{i}}\nabla P\;i=w,\;g,\;v$$

#### 2.1.4. Energy Balance

Assuming all the components, including solid, water, and gas, are all in energy equil ibrium, the microscopic energy balance can be expressed as follows [15]:

(14) $$\rho_{eff}c_{p,eff}\frac{\partial T}{\partial t}+\underset{i=w,v,a}{\sum }\left(n_{i}\nabla \left(C_{p,i}T\right)\right)=\nabla \left(k_{eff}\nabla T\right)-{\;\lambda \dot{I}+Q}_{mic}$$

where the evaporation rate (*İ*, in a unit of mol·m^−3^·s^−1^) and RF energy absorption rate ($Q_{mic}$, in a unit of J·s^−1^) are all functions of time and space; *λ* is the latent heat of vaporization (J·kg^−1^).

Effective parameters, including density (*ρ_eff_*), specific heat (*c_p,eff_*), and thermal conductivity (*k_eff_*), are used for describing the volumetric properties of food material [15]:

(15) $$\rho_{eff}=\left(1-\;\emptyset \right)\rho_{s}+\emptyset {(S}_{w}\rho_{w}{+S}_{g}\rho_{g})$$

(16) $$c_{p,eff\;}{=m}_{s}c_{p,s}{+m}_{w}c_{p,w}{+m}_{g}{(m}_{a}c_{p,a}{+m}_{v}c_{p,v})$$

(17) $$k_{eff}=\left(1-\;\emptyset \right)k_{s}+\emptyset {(S}_{w}k_{w\;}{+S}_{g}\left(w_{v}k_{v}{+w}_{a}k_{a}\right))$$

where *ρ_eff_* is the effective density (m^3^·kg^−1^), *c_p,eff_* is the effective heat capacity (kJ·kg^−1^·K^−1^), and *k_eff_* is the effective thermal conductivity (W·m^−1^·K^−1^).

#### 2.1.5. Dielectric Properties

The dielectric properties of food are significantly influenced by water content in a drying process. A developed equation, namely the “Landau and Lifshitz, Looyenga equation (LLLE)”, was used to estimate dielectric properties of potato samples with various water content and at different temperatures [23]:

(18) $$\varepsilon^{\frac{1}{3}}=\underset{i=s,w,g}{\sum }v_{i}\varepsilon_{i}^{\frac{1}{3}}$$

where *v_i_* is the volume fraction of phase *i*, and the $\varepsilon_{i}$ is the dielectric properties of phase *i*.

#### 2.1.6. Permeability

The permeability of porous materials is influenced by the properties of the pores inside the material and the transport properties of the fluid phase. The total permeability of a material is the product of its intrinsic permeability (which depends on structural parameters such as porosity) and relative permeability (which depends on the relative saturation of the fluid phase) as follows [16]:

(19) $$k_{tot,i}=k_{in,i}k_{r,i}\;\;\;\;\;\;i=w,\;g$$

where *k_tot,i_* is total diffusivity of phase *i* (m^2^); *k_in,i_* is intrinsic permeability of phase *i* (m^2^); *k_r,i_* is relative permeability of phase *i* (–).

The intrinsic permeability of dry solid is difficult to obtain theoretically and experimentally [16]. Thus, in this study, the intrinsic permeability of the potato solid was estimated as $1\times 10^{-16}$ m^2^ based on a trial-and-error method. An assumed diffusion coefficient was firstly assumed and brought into the model to calculate the drying curve, and the result was compared with the experimental drying curve. If the mean square error was higher than 0.05, the diffusion coefficient value was modified accordingly, and the above process was repeated until the error met the requirement. As reported, the range of intrinsic permeability values of raw potatoes was $10^{-15}-10^{-17}$ m^2^ [16].

The intrinsic permeability of gas is [24]:

(20) $$k_{in,g\;}{=k}_{in,w}(1+\frac{{0.15k}_{in,w}^{-0.37}}{P})$$

The relative permeability of water is [25]:

(21) $$k_{r,w\;}=\left\{\begin{array}{cc} {\left(\frac{S_{w}-0.09}{1-0.09}\right)}^{3}, & {\;S}_{w}\;>\;0.09\\ 0 & {\;S}_{w}\;<\;0.09 \end{array}\right.$$

where *S_w_* is the saturation ratio of water (–).

The relative permeability of gas is [25]:

(22) $$k_{r,g}=\left\{\begin{array}{cc} 1{-1.1S}_{w}, & \;{\;S}_{w}\;>\;0.09\\ 0 & {\;S}_{w}\;<\;0.09\; \end{array}\right.$$

#### 2.1.7. Initial and Boundary Conditions

The voltage of the top electrode was initially set according to the calculated results. The value was tuned with a trial and error method and finalized when predicted results were matched with the experimental temperature pattern and evaporation rate of samples [26]. The bottom electrode was set as grounded (*V* = 0). The metal enclosure of the RF cavity was regarded as the ideal conductor and was defined as electrical insulation (∇*E* = 0). Other initial and boundary conditions are listed in Table 1.

When the water vapor on the surface of the potato reaches saturation, the water inside the potato will transfer directly to the surface in liquid form. The boundary flux of water can be expressed as [16]:

(23) $${\dot{J}}_{\left.\bar{n},w\right|surf}{=h}_{m}\emptyset S_{w}\left(\rho_{v}-\rho_{v,amb}\right){+c}_{w}{\bar{v}}_{\bar{n},w}{\;(\mathrm{when}\;S}_{w}=1)$$

where ${\dot{J}}_{\left.\bar{n},w\right|surf}$ is the boundary flux of water a phase (kg·m^−2^·s^−1^); *h_m_* is the mass transfer coefficient (m·s^−1^); $\rho_{v}$ is the vapor density on the sample surface (kg·m^−3^); $\rho_{v,amb}$ is the vapor density in ambient air (kg·m ^−3^); ${\bar{v}}_{\bar{n},w}$ is the water velocity on the sample boundaries (m·s^−1^).

Water vapor can be blown out of the surface if the internal pressure within the food is too high. Thus, the boundary flux of water vapor was set as [16]:

(24) $${\dot{J}}_{\left.\bar{n},v\right|surf}{=h}_{m}\emptyset S_{g}\left(\rho_{v}-\rho_{v,amb}\right){+c}_{g}{\bar{v}}_{\bar{n},g}$$

where ${\dot{J}}_{\left.\bar{n},v\right|surf}$ is the boundary flux of the vapor phase (kg·m^−2^·s^−1^).

The total amount of heat transfer on the sample surface is composed of the following four items: heat convection between food and the surrounded air, the heat loss due to water evaporation on the food surface, the heat loss due caused by water vapor leaving the food surface, and the heat loss due to water loss as mass transfer. The accumulated heat was calculated as [16]:

(25) $${\left.q\right|}_{surf}{=h}_{t}\left(T_{surf}-T_{amb}\right)\;{-\;\lambda \dot{J}}_{\left.\bar{n},w\right|surf}\;-\;\sum_{i=w,v}\left({\dot{J}}_{\left.\bar{n},i\right|surf}\right)C_{p,v}T_{surf}\;-\;c_{w}{\bar{v}}_{\bar{n},w}C_{p,w}$$

where ${\left.q\right|}_{surf}$ is the heat transfer on the sample boundary (W·m^−2^); *h_t_* is the convective heat transfer coefficient (W·m^−2^·K^−1^); *T_surf_* is the temperature of the sample surface (K); *T_amb_* is the temperature of ambient air (K).

#### 2.1.8. Computer Simulation

**Figure 2:**
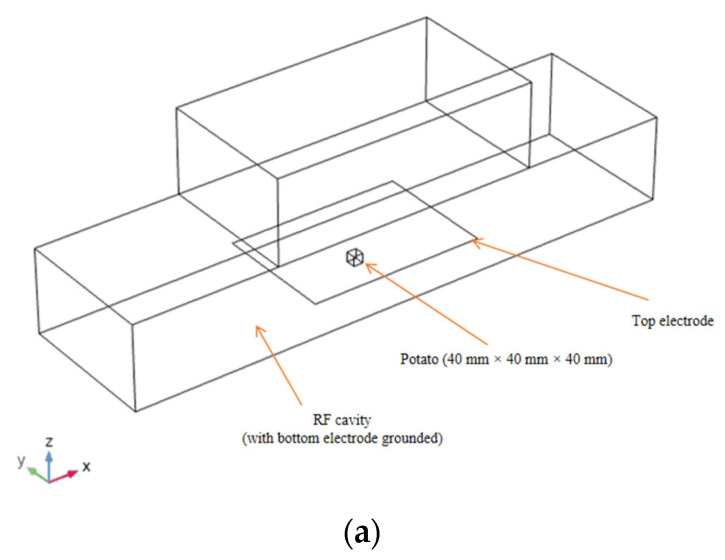

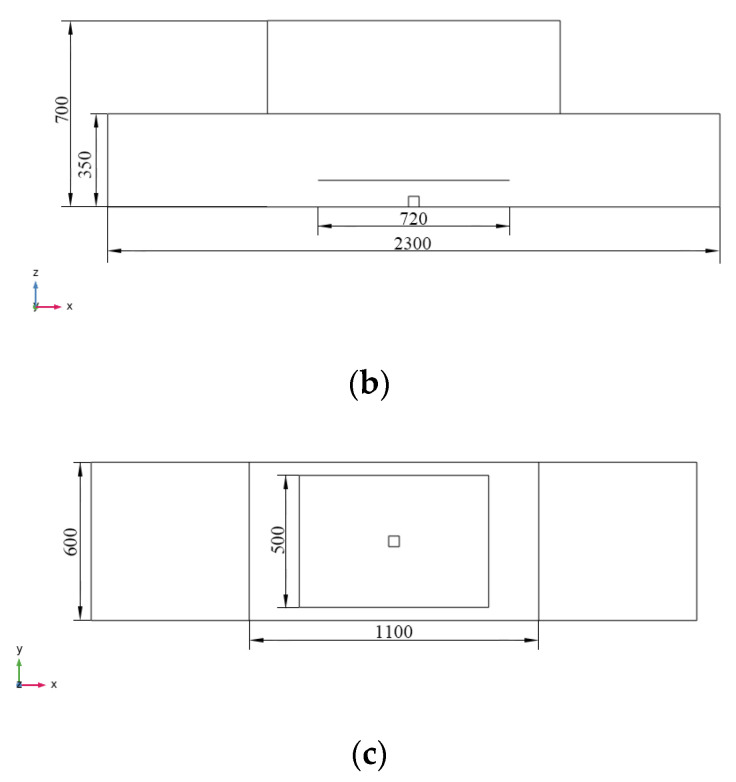
Geometric model of RF drying heater and placement of potato: (**a**) layout, (**b**) front view, and (**c**) top view (all dimensions are in mm).

There modules including “electric current (AC/DC)”, “heat transfer in the solid”, and “dilute phase flow” were selected and coupled with the parameter of water content and temperature in COMSOL Multiphysics^®^ (COMSOL Multiphysics 5.2, COMSOL Inc., Burlington, MA, USA). The scheme of the RF heater is described in Figure 2. A potato cube (40 × 40 × 40 mm) was placed at the center of the bottom electrode, with an electrode gap between the top and bottom electrode of 100 mm. After geometry creation, the material properties and appropriate initial/boundary conditions were set up for all domains according to experimental conditions, which was discussed in Section 2.1.7. Tetrahedral mesh was applied to all domains. The mesh size was selected as “extremely fine” for the potato sample, and the maximum size of the mesh was set as 0.00242 m. The determination of mesh size was based on the comprehensive consideration of mesh convergence criteria and saving computing resources. That is, the maximum temperature difference between the temperature–time curves of the sample before and after continuous refinement of the mesh was less than 1%. The mesh size before refinement was selected considering the computational resources and the accuracy of calculation. The total meshes include 152,638 domain elements, 7699 boundary elements, and 495 edge elements. The Multifrontal Massively Parallel Solver (MUMPS) was selected with the time step set as 0.001 s, and the backward difference iteration method was adopted for solving the model. The temperature field, the moisture content distribution, the water vapor concentration, and the temperature–time history of the sample center were obtained, and the changes of each value in the drying process were compared, respectively. The simulation process was performed on a Dell workstation with two dual-core Intel Xeon CPU 2.60 GHz processors and 128 GB RAM with the Microsoft Windows Server (Microsoft Corporation, Redmond, WA, USA) 2012 R2 standard operating system installed. The total computing time was 572 min.

### 2.2. Experimental Validation

#### 2.2.1. Material Preparation

Fresh potatoes (*Solanum tuberosum* L., locally planted in Shanghai) without dents and mold areas were purchased from a local supermarket (Lingang, Shanghai, China). Potatoes were equilibrated in a 10 °C, clean, and dry environment for storage for 24 h. Before the experiment, three potatoes were rinsed, peeled, and dried with tissue papers. From each potato, a cube sample was cut from the center with an identical dimension of 40 × 40 × 40 mm and weight of 70 g for drying experiments.

#### 2.2.2. Dielectric Properties Determination

Dielectric properties of the food sample were necessary input parameters in the modeling process, which were influenced significantly by the temperature variation and moisture content of the samples. As the most popular dielectric properties measuring technique, the transmission line method and open-coaxial probe method were used to measure the dielectric properties of food. However, few measurement techniques were found to be successful for measuring porous materials [32,33]. It was due to the non-uniform geometry of the porous material, the uneven pore size distribution, and the incomplete contact between the material surface and the dielectric probe [23]. Thus, the dielectric properties of potato powder and water were measured, respectively, and an estimation equation, namely “Landau and Lifshitz, Looyenga equation (LLLE)” was employed to predict the dielectric properties of potatoes at different water contents and different temperatures following Equation (18) in Section 2.1.5. Since the dielectric properties of potato powder change little with the temperature at 27.12 MHz in the drying temperature range (12~50 °C), temperature effect was ignored in the dielectric properties measurement of potato powders.

Potato cubes (100 g) were placed in a hot-air drying chamber (GZX-9076 MBE, Boxun, Shanghai, China) at 110 °C and sampled every 2 h until reaching a constant weight (weight change between two measurements was <0.1 g). The dried potato samples were grounded to potato powder with an automatic grinder (JYS-M01, Jiuyang, Chengdu, China) for dielectric properties determination. Dried potato powders were placed into a self-designed sample holder until full, and an open-ended probe (Agilent N1501A, Agilent Technologies Inc., San Jose, CA, USA) connected to a network analyzer (Agilent E5071C, Agilent Technologies Inc., San Jose, CA, USA) was utilized for measurement. The detailed dielectric properties measurement procedure can be found in the literature [34]. The experiment was replicated three times, and an average value was used in the modeling process.

#### 2.2.3. RF Drying Experiment

A 12 kW, 27.12 MHz, free-running type RF heater (GJD-12A-27-JY, Huashijiyuan High-Frequency Equipment, Cangzhou, Hebei, China) was used for model validation. The electrode gap was set as 100 mm. Three potato cubes were placed at the center of the bottom electrode with a space of 100 mm. Before the drying experiment, the initial temperatures of all potato sample cubes were verified with an infrared camera (FLIR A655sc, Wilsonville, OR, USA) as 10.0 ± 1.0 °C. The initial weights of potato samples were also measured. The center temperatures of potato samples during drying were recorded by inserting fiber optic sensors (HQ-FTS-D1F00, Heqi guangdian, Xi’an, China) into the geometrical center. Sample weights were measured, and temperature profiles of sample surfaces were captured every 5 min throughout the RF drying process, which required a temporary pause of the process. When treatment was suspended, the sample was quickly taken to an infrared camera for obtaining thermal images, then transferred to a electronic balance for weighing, and eventually put back into the original position of the RF cavity to continue drying. This process was completed within 10 s to minimize the heat loss. The drying process lasted for 720 min until the sample moisture content reached 0.35 g·cm^−3^. The experiment was replicated three times.

The dry basis water content in potato samples was calculated as follows [35]:

(26) $$X_{i}=\frac{w_{i}-w_{end}}{w_{end}}$$

where *X_i_* is the water content in potato samples at the time *i* (g·g ^−1^), *w_i_* is the potato weight at the time *i* (g), and the *w_end_* is the absolute dry weight of potato samples (g).

## 3. Results and Discussion

### 3.1. Electromagnetic Field Distribution

**Figure 3:**
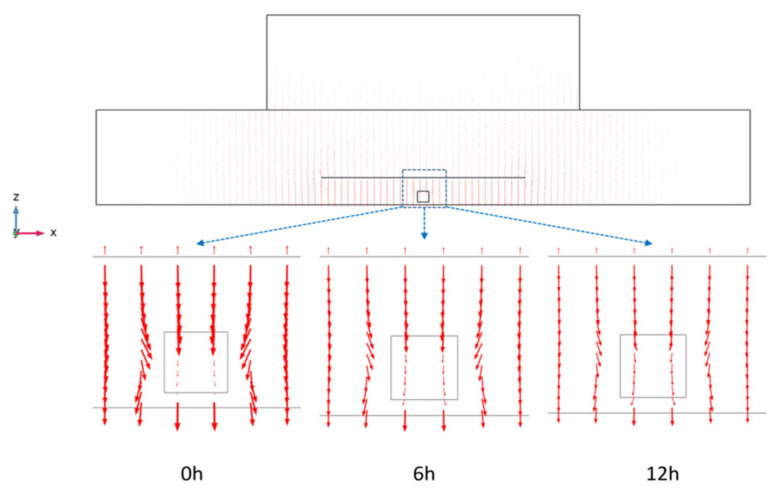
Electric field distribution in the central section of the radio frequency heater cavity (*y* = 300 mm).

The visualized electromagnetic field distribution within the potato sample is plotted at the time of 0, 6, and 12 h for comparison in Figure 3. A certain angle of deflection of the electric field was observed at the edges, especially the corners of the potato cube sample, throughout the drying process, which is usually the main cause of edge heating. This was possibly attributed to the sample size being smaller than the top electrode, and the electric field was deflected at the edges at the surface of potato sample [36]. The deflection angle was reducing as the drying process continued, which indicated that the RF drying process led to faster water evaporation at the corners and enhanced the electric field intensity at the edges.

### 3.2. Temperature Distribution

**Figure 4:**
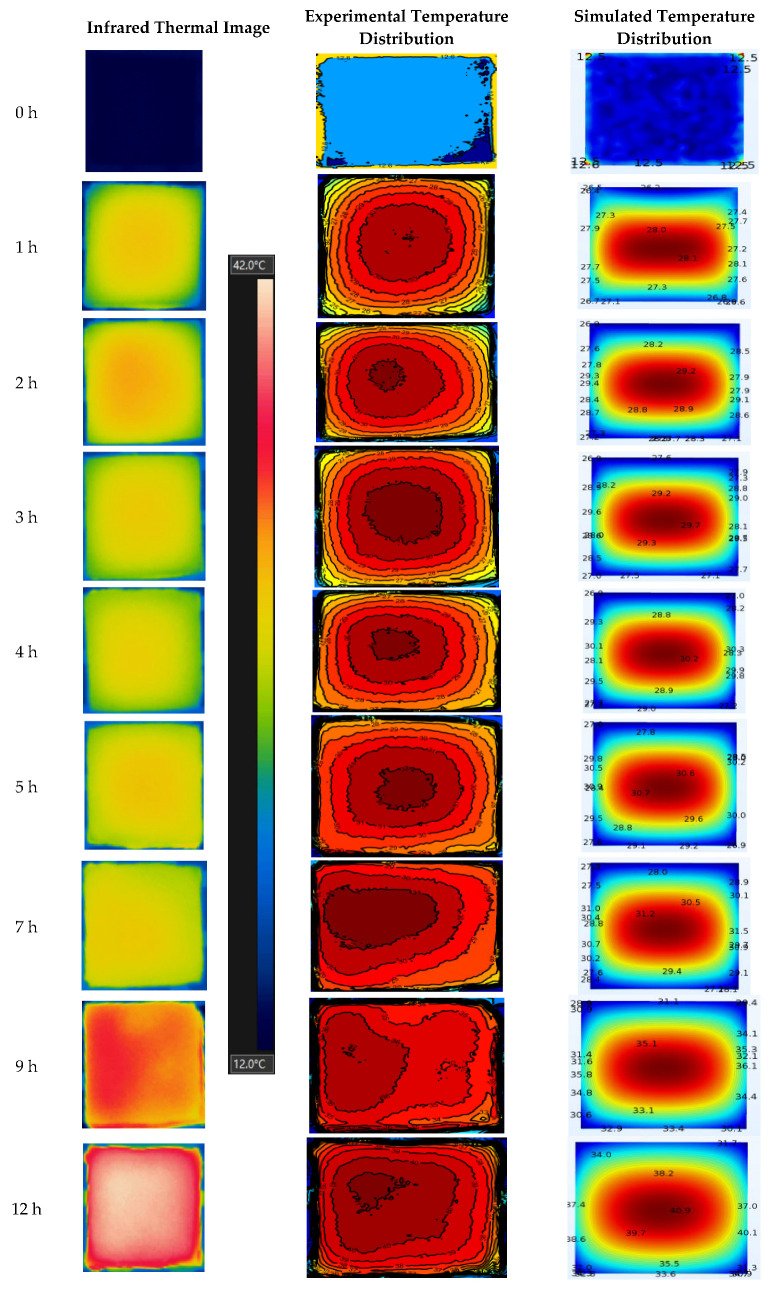
Experiment and simulated temperature profiles of potatoes after RF drying.

Figure 4 shows the temperature distribution at the potato cube surfaces from both computer simulation and experiment during RF drying. Results showed a similar distribution pattern between simulation and experiment results. The center temperature increased faster than that at the edges, which was possibly caused by a higher moisture content at the centers, with the electromagnetic field focused on this zone, and resulted in a higher temperature. This is the moisture auto-balancing advantage for RF drying since it prevents the sample from overheating [19]. Similar results were also reported by Wang et al. [37] and Zhou et al. [38]. Within the 1st h of drying, a significant temperature increase at the sample surface was found, and the temperature difference between simulation and experiment was less than 2.6 °C. At this stage, results demonstrated that the simulated temperature distribution pattern matched the experimental one well. Within the 1~7 h of drying, the surface temperature increased by only ~2 °C. The energy absorption was not reflected in the temperature increase because of water evaporation [38]. In general, the experimental results were also in good agreement with the simulation results. However, the hot region started moving to the left boundary from the center, while the simulation result was still in the center of the sample surface as shown in 7 h temperature diagrams. This might be due to uneven water transfer in the actual drying process caused by uneven shape or accidental thermal-runaway phenomena. After 7 h treatment, sample temperature started to increase significantly again, and the drying process entered the falling rate period, which indicated insufficient water in the sample for continuous evaporation [39]. The hot spot area continued to move to the left boundary of the sample in this stage, while the simulation results were still in the central position. The discrepancy between simulation and experimental results was because the measurement errors of material properties were exaggerated during drying experiments.

**Figure 5:**
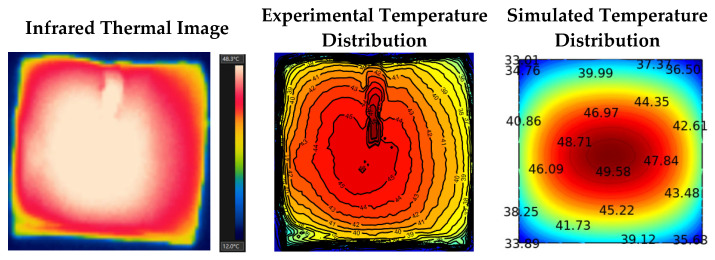
Temperature distribution on the cross-sectional (the *zx*-section at the center) surface of potato sample from simulated and experimental results.

After the drying experiment, the potato sample was quickly cut into halves with a thin blade, and the temperature profile of the cross-sectional surface was captured with an infrared thermal imager. Figure 5 compares the temperature distribution on the sample cross-sectional surface between the experimental and the simulated results. The internal temperature of the sample was reduced to a certain extent due to cutting. The temperature drop was ~3.2 °C from the comparison of fiber optic sensor reading (51.2 °C) and the infrared camera capture at the center point (48.0 °C). An interesting phenomenon was observed: the temperature at the center of the sample where the fiber sensor was inserted was higher than that of the other areas of the sample, as shown in Figure 5. The possible reason is that there was a little gap between the fiber optic sensor and the potato sample during insertion, resulting in a localized heating phenomena.

**Figure 6:**
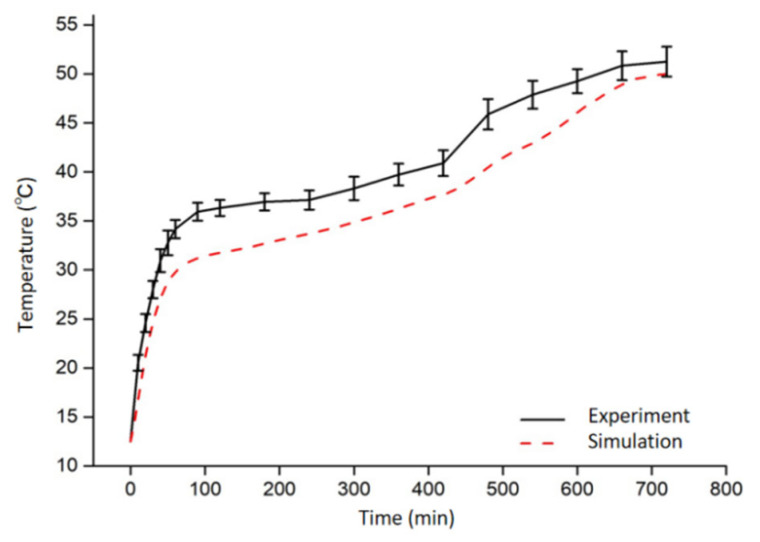
Time–temperature curve of the center point of potato during RF drying from both experiment and simulation.

The time–temperature history curves of the sample center for both experiment and simulation results are shown in Figure 6. The curve could be divided into three stages: In stage I (0–1 h), the temperature increased rapidly from 12.0 to 34.0 °C, this could be attributed to the electromagnetic energy was mainly used for temperature elevation of samples; in stage II (1–7 h), the temperature of sample increased slowly by 4.0 °C, which was due to the usage of energy for water evaporation; in stage III (7–12 h), the sample temperature increased rapidly from 38.0 to 51.2 °C due to the water removal, and the specific heat capacity of the sample decreased with the water loss [40]. The experimental results were consistent with the simulated results in the trend. The temperature of the experimental value was higher than that of the simulated value, and the temperature difference between the two was < 5.5 °C. This may be due to the pore shrinkage that occurred within the volume of sample during the drying process, which was not considered in the simulation. Drying would result in surface hardening and volume shrinkage due to the loss of water. As the water transfer from the internal part of the sample to the surrounding air was limited, a relatively high water content and higher dielectric loss factor in the sample would be retained and ultimately result in a high temperature inside the sample volume [41].

### 3.3. Moisture Migration

**Figure 7:**
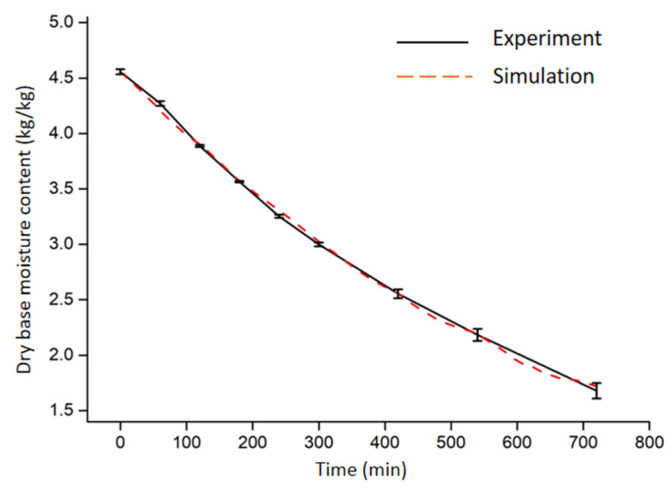
Moisture content of potato samples during RF drying from both simulation and experiment.

The removal rate and distribution of moisture content distribution are important parameters in evaluating a drying process. Figure 7 shows the reduction amount of water content (d.b.) with time from both experimental and simulation results. During the drying process, the water content showed a linear decline from both experiment and simulation, and the drying rate had little discrepancy. During the 12 h drying period, the experimental and simulated values were highly consistent, and the simulated error of moisture content (d.b.) was less than 0.18. During the RF drying process, the drying rate was constant in the previous part, then gradually decreased to the end. This is in line with an earlier report for carrot slices dried with combined microwave and vacuum treatment [39]. The initial dry basis moisture content of the potato was 4.56 (d.b.) and decreased to 1.75 (d.b.) after 12 h drying. This indicates that RF drying is effective for potatoes.

**Figure 8:**
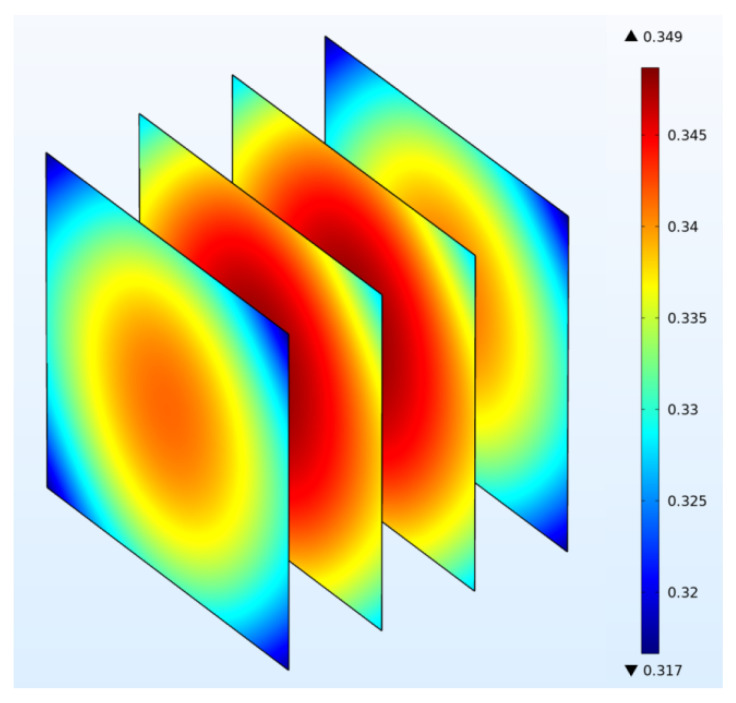
Distribution of water concentration (mg·cm^−3^) in potato samples from simulation (*t* = 12 h).

**Figure 9:**
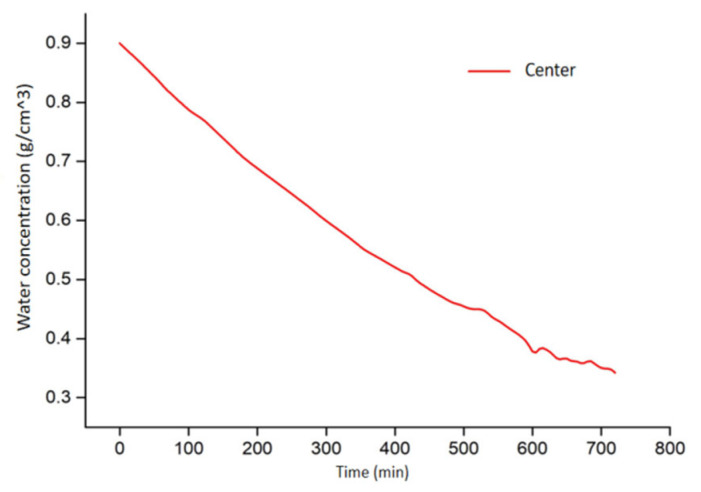
Water concentration at the center of potato samples during RF drying.

Figure 8 shows the water distribution inside the sample from modeling after 12 h of drying. The water concentration in the sample is non-uniform, and the maximum difference of water concentration within the volume is 0.03 g·cm^−3^. The moisture distributions within the sample corresponded to the temperature–time history inside the sample during drying: the central area had a higher water concentration than the corner. A similar water concentration distribution pattern was also reported for kiwifruit slices in RF–vacuum drying [15,38]. Figure 9 shows that the moisture content of samples decreases with increased drying time, and the drying rate decreases at the final drying period (600–720 min). The main reason is that the moisture within the sample decreased at the falling rate period, which leads to a slower heating rate with the decrease of dielectric loss factor [42]. The initial water concentration at the sample center was 0.90 g·cm^−3^ and decreased to 0.35 g·cm^−3^ after 12 h drying.

### 3.4. Vapor Migration

**Figure 10:**
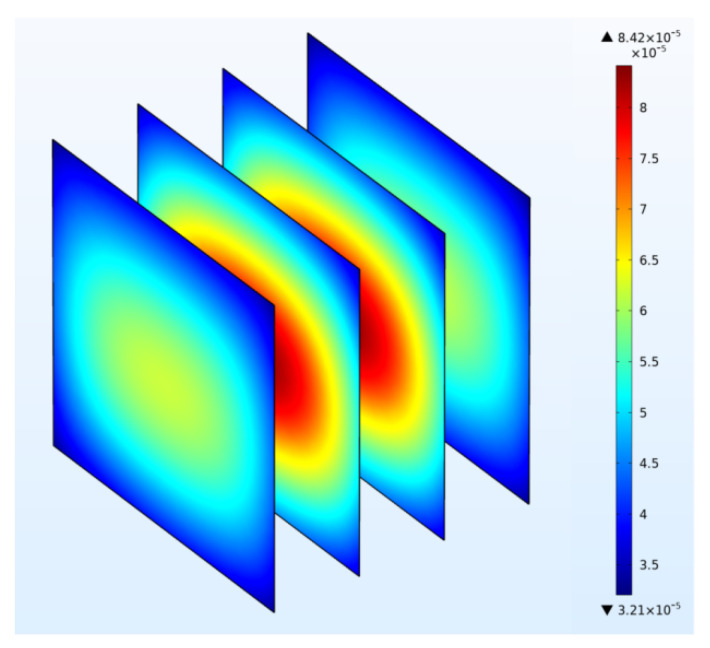
Distribution of vapor concentration (mg·cm^−3^) in potato samples during RF drying from simulation (*t* = 12 h).

**Figure 11:**
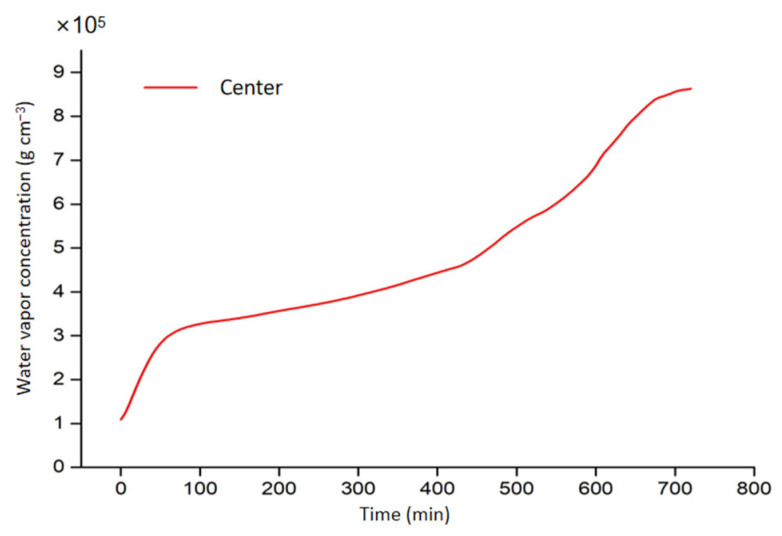
The water vapor content in potato samples with RF drying duration from simulation.

Figure 10 and Figure 11 show the water vapor concentration distribution in potato samples after drying and the water vapor concentration at the center point of the potato sample during drying over time from modeling. The distribution of water vapor concentration is related to the distribution of both the temperature and water content within the sample since higher temperature led to a higher evaporation rate of water vapor [43]. Results indicate that the water vapor concentration at the sample center is greater than that at the corners because water vapor increased as temperature increased, and a pressure gradient from center to corner would guarantee the mass transfer from the sample to the surrounding area in the drying process. It can be seen from Figure 11 that the trend of water vapor concentration variation at the sample center corresponds to the change of temperature at the same spot, as shown in Figure 5. This indicates the temperature and water vapor concentration in the center of the sample increase as the electromagnetic energy continually converts to heat energy. This is due to the selective heating mechanism of RF heating: the larger moisture content results in a higher energy localization, which facilitates the drying process [14]. Moreover, since the speed of water migration from the inside to the surface of the sample was relatively stable, the variation of the evaporation speed was reflected on the concentration of water vapor.

From comparison, the developed model was effective and reliable in predicting temperature and moisture distribution in a model food system.

## 4. Conclusions

In this study, a numerical model of RF drying was established and solved by coupling electromagnetic heating, heat transfer, and mass transfer in a potato cube sample, and the temperature, moisture content, and vapor variation and distribution in the sample in RF drying were described. The prediction model was proven to be reliable and effective through experiments. The distribution of water concentration and water vapor concentration corresponds to the temperature distribution. The results of RF drying potato showed a relatively constant drying rate throughout the RF drying process; thus, it could be used as a supplemental method for traditional drying processes to improve the drying rates and quality of products. The numerical model developed in this research could be used as a tool to explore the mechanism of RF drying processes and also to provide a convenient and economic method for food drying.

To promote industrial applications of RF drying, it is suggested that future research could focus on the following directions:

(1) One of the major challenges of RF heating is non-uniform heating, which results in non-uniform quality for dried products. Products with irregular geometry need particular attention in RF drying since the drying uniformity was significantly influenced by sample shapes and sizes.

(2) The properties of fluids and coefficients in mass and heat transfer, such as porosity, viscosity, permeability, diffusion coefficient, etc., had significant influences on heat and mass transfer processes in the RF drying process. To improve the accuracy of the simulation, sensitivity analyses of these parameters were needed, and the significance of influence of each parameter should be evaluated.

(3) It would be necessary to broadly investigate the combination of RF drying with some other technologies (freeze drying, vacuum, cold air, etc.) to further improve the quality of the final dried product. In a combined drying process, the hurdle effect needs to be explored and emphasized for optimizing the processes.

## Figures and Tables

**Table 1:** Input parameters used in simulation.

Symbol	Parameter	Value	Unit	Reference
*f*	Frequency	27.12	MHz	
*V*	Electrode voltage	2750	V	
Solid				
$\varepsilon_{s}^{\prime }$	Dielectric constant	2.4	-	
$\varepsilon_{s}^{\prime \prime }$	Dielectric loss factor	0.4	-	
$k_{s}$	Thermal conductivity	0.21	W·m^−1^·K^−1^	[27]
$\rho_{s}$	Density	1528	kg·m^−3^	[28]
$C_{ps}$	Specific heat	1650	J·kg^−1^·K^−1^	[27]
Water				
$\varepsilon_{w}^{\prime }$	Dielectric constant	−0.2833T + 80.67 ^a^	-	[29]
$\varepsilon_{w}^{\prime \prime }$	Dielectric loss factor	0.05T + 20 ^a^	-	[29]
$k_{w}$	Thermal conductivity	0.59	W·m^−1^·K^−1^	[27]
$\rho_{w}$	Density	998	kg·m^−3^	
$C_{pw}$	Specific heat	4182	J·kg^−1^·K^−1^	[16]
$\mu_{w}$	Viscosity	$0.998\times 10^{3}$	Pa s	[16]
$k_{in,w}$	Intrinsic permeability	$1\times 10^{-16}$	$m^{2}$	
$k_{r,w}$	Relative permeability	Equation (21)		
Water vapor				
$k_{v}$	Thermal conductivity	0.026	W·m^−1^·K^−1^	[27]
$\rho_{v}$	Density	Ideal gas ^b^	kg·m^−3^	
$C_{pv}$	Specific heat	2062	J·kg^−1^·K^−1^	[16]
Air				
$k_{a}$	Thermal conductivity	0.026	W·m^−1^·K^−1^	[27]
$\rho_{a}$	Density	Ideal gas ^b^	kg·m^−3^	
$C_{pa}$	Specific heat	1006	J·kg^−1^·K^−1^	[27]
Gas (air + water vapor)				
$\mu_{g}$	Viscosity	$1.8\times 10^{-5}$	Pa s	[16]
$k_{in,g}$	Intrinsic diffusivity of gas	Equation (20)		[24]
$k_{r,g}$	Relative diffusivity of gas	Equation (22)		
Other				
$D_{w,cap}$	Capillary diffusion rate	$10^{-16}\exp(-2.8+2M)$ ^c^	m^2^·s^−1^	[21]
$P_{v,eq}$	Equilibrium vapor pressure	$P_{\mathrm{sat}}\exp({-0.0267M}^{-1.656}$ $+{0.0107e}^{-1.287M}M^{-1.513}\ln\left(P_{\mathrm{sat}}\right))$ ^c, d^		
$h_{t}$	Convective heat transfer coefficient	20	W·m^−2^·K^−1^	[30]
$h_{m}$	Mass transfer coefficient	$2\times 10^{-7}$	m·s^−1^	[31]
$\rho_{a}$	Density of air	Ideal gas ^b^	kg·m^−3^	
$D_{bin}$	Diffusion rate of water vapor in air	$\frac{2.13}{P}{\left(\frac{T}{273}\right)}^{1.8}{{(S}_{g}\emptyset )}^{3-\emptyset }/\emptyset$ ^a, d^	m^2^·s^−1^	
$P_{amb}$	Atmospheric pressure	${1.01\times 10}^{5}$	Pa	
*λ*	Latent heat of evaporation	${2.26\times 10}^{6}$	J·kg^−1^	
$K_{evap}$	Evaporation rate constant	1000	s^−1^	[21]
∅	Porosity	0.88		
$c_{w,0}$	Initial water concentration	797.4	kg·m^−3^	
$w_{w,0}$	Mass fraction of water vapor	0.026		
*T*_0_	Initial temperature of sample	285.65	K	
*T_amb_*	Ambient temperature	283.15	K	

^a^ all the temperatures (T) in the table are in a unit of °C; ^b^ all the ideal gas densities in the table are 1.205 kg·m^−3^; ^c^ all the moisture contents (M) in the table are in a unit of (kg water/kg dry solid); ^d^ all the pressures in the table are in a unit of Pa.

## Data Availability

The data presented in this study are available in this article.

## Nomenclature

*V*	Voltage (V)
*V_i_*	Volume of component *i* (m^3^)
*f*	Frequency (Hz)
*Q*	RF power conversed to thermal energy (W·m^−3^)
*E*	Electric field intensity (V·m^−1^)
*c_i_*	Concentration of a fluid phase, *i* (mol·m^−3^ )
*n_i_*	Mass flux per unit volume of a fluid phase, *i* (mol·m^−3^ )
*İ*	Rate of evaporation (mol·m^−3^·s^−1^)
*P*	Vapor pressure (Pa)
*P_w_*	Capillary pressure (Pa)
*P_c_*	Net pressure (Pa)
*D_w,cap_*	Capillary diffusivity of a fluid phase, *i* (m^2^·s^−1^)
*k_in,i_*	Intrinsic permeability of a fluid phase, *i* (m^2^)
*k_r,i_*	Relative permeability
*k_tot,i_*	Total permeability of a fluid phase, *i*
*D_bin_*	Diffusion rate of water vapor in air (m^2^·s^−1^)
*M*	Moisture content (kg water/kg dry solid)
*M_a_*	Molecular weight of air
*M_v_*	Molecular weight of vapor
*X_i_*	Water content in potato samples at the time *i* (g·g^−1^)
*w_i_*	Potato weight at the time *i* (g)
*w_end_*	Absolute dry weight of potato samples (g)
$\bar{\nu }$	Velocity (m·s^−1^)
*S_i_*	Saturation of fluid phase, *i* (−)
*C_p,i_*	Specific heat of component *i* (J·kg^−1^·K^−1^)
*P_v,ep_*	Equilibrium vapor pressure (J·kg^−1^)
*K_evap_*	Evaporation rate constant (s^−1^)
*h_t_*	Heat transfer coefficient (W·m^−2^·K^−1^)
*h_m_*	Mass transfer coefficient (m·s^−1^)
*P_amb_*	Atmospheric pressure (Pa)
*t*	Time (min)
Δ*t*	Time interval (min)
*T*	Temperature (°C)
*R*	Universal gas constant (kJ·kmol^−1^·K)
*k_i_*	Thermal conductivity of component *i* (W·m^−1^·K^−1^)
Greek symbols	
*μ_i_*	Viscosity of component *i* (Pa·s)
*ε*_0_	Free space permittivity (8.86 × 10 ^−12^ F·m^−1^)
*ε′*	Relative dielectric constant (−)
*ε″*	Relative dielectric loss factor (−)
*σ*	Electrical conductivity (S·m^−1^)
*ϕ*	Porosity (−)
∇	Gradient operator
*λ*	Latent heat of vaporization (J·kg^−1^)
*ρ_i_*	Density of component *i* (kg·m^−3^ )
Subscript	
a	Air
s	Solid
w	Water
f	Fluid
g	Gas
v	Vapor
eff	Effective
surf	Surface
amb	Ambient air
eq	Equilibrate
r	Relative
in	Intrinsic
0	Time *t* = 0
tot	Total
sat	Saturate
